# Pellagra as It Occurs in Buffalo and Vicinity

**Published:** 1918-06

**Authors:** Grover W. Wende

**Affiliations:** Buffalo, N. Y.


### BUFFALO MEDICAL JOURNAL

Yearly Volume 73 JUNE, 1918 Number 11

### ORIGINAL ARTICLES

**The right is reserved to decline papers not dealing with practical medical and surgical subjects, and such as might offend or fail to interest readers. Contributors are solely responsible for opinions, methods of expression and revision of proof.**

### Pellagra as it Occurs in Buffalo and Vicinity

By GROVER W. WENDE, M. D., Buffalo, N. Y.

The sudden, widespread and remarkable frequency of the occurrence of indigenous Pellagra in the United States constitutes a unique chapter in the medical history of this country. Prior to 1907, Pellagra was very seldom recognized in America. The isolated cases reported before 1907 occurred in foreign born individuals who either came to the United States with the disease in full efflorescence or, subsequently developed it.

In 1864, Dr. John P. Gray, first reported the occurrence of Pellagra in the State of New York and the history of Pellagra in the Empire State begins with the publication of this case. The patient was a foreigner and an inmate of the Utica State Hospital. Little more was heard of Pellagra in New York until 1882, when Dr. Samuel Sherwell of Brooklyn reported its occurrence in an Italian sailor. He reported a second case of this disease in 1902, also in an Italian sailor. With the possible exception of two or three cases appearing in other parts of North America these New York cases were the only ones occurring in the United States reports of which were published before 1907. In 1907, Pellagra cases began to be reported in startling numbers from the Southern States and especially from institutions for the insane. Since then the disease has appeared with ever increasing frequency in all the states, in some even taking on the aspect of an epidemic.

The first cases of Pellagra indigenous to the State of New York were reported by both Caccini and the author. Subsequently, indigenous cases were reported by Robbins, Spangenthal, MacKee, Wise and Lautman, Winfield, and Potter.

That these do not represent all the indigenous cases is shown by the statistics of the New York State Department of Health and that of the City of New York wherein are recorded in 1912 one death, in 1913 four deaths, in 1914 sixteen deaths, in 1915 ten deaths, in 1916 twenty-seven deaths, and in 1917 twenty-seven deaths, either primarily or secondarily due to Pellagra. Outside of the City of New York fifteen of the twenty-six pellagrins dying and reported in 1916 and 1917 were inmates of state or other hospitals.

Pellagra prevails with greater frequency among women than among men. No period of life seems to be exempt from its ravages although it occurs most commonly during adult life,—between the ages of twenty and fifty. Inhabitants of rural districts seem to contract the disease with much greater frequency than those living in towns. The poor and ill-nourished are particularly prone to Pellagra, although in the Southern States the well-to-do are frequently attacked.

### SYMPTOMATOLOGY.

The symptomatology of Pellagra is best considered under three heads. In order of importance for diagnosis these are (1) Skin manifestations, (2) Alimentary tract manifestations, and (3) Nerve and Mind manifestations.

The cutaneous eruptions of Pellagra are highly characteristic. At present they are the principal determining factors in making a positive diagnosis, nevertheless Pellagra may be present without skin lesions. The skin manifestations frequently occur in so-called "stages,"—erythema, edema, pigmentation, desquamation, bullae formation, ecchymoses, and pyogenic infection. However, these "stages" are but manifestations of the degree of activity of the disease process and of its progress; there is no sharply defined "stage" for all may be present at any one time and they shade into each other.

The eruption begins suddenly nearly always in the early days of spring as an erythema usually symmetrically disposed on the backs of the hands. The arrangement and location of the skin lesions of Pellagra are particularly characteristic. Although now and then involving parts of the body protected from sunlight by clothing or much hair the disease attacks only those portions of the person usually uncovered, unprotected. The erythema very rapidly assumes a brilliant hue. A moderate amount of swelling of the skin is usually present from the very first. The outlines of the diseased patches are very sharply defined, much more so than those resulting from simple sunburn. As the disease advances the color of the lesions deepens to a reddish-brown or even to a chocolate hue. After ten or more days, desquamation begins in the lesions and a rough and scaly surface is produced. Where the disease process is very active, vesicles and bullae develop upon the erythematous base in which are often found ecchymoses. Secondary infection with the ordinary pyogenic organisms may follow. As the vesicles and bullae desiccate, thick crusts are formed and exfoliation of large masses of epithelium takes place, leaving behind raw, red surfaces. If the process is less acute, the skin surfaces are thick, hard, rough, scaly and often pigmented; the elasticity of the skin in these areas is lost, producing fissures and an exaggerated width and depth to the normal lines of the skin.

Upon the backs of the hands are found the most striking and typical cutaneous manifestations of Pellagra. The lesions affect both hands in a notably symmetrical way. When fully developed they spread from the radial to the ulnar side of the dorsum of the hands even so as to include the fingers and knuckles; the palmer surfaces are attacked with extreme rarity, while lesions may involve a small triangular area on both the extensor and flexor surfaces of the wrists. The full typical involvement is often spoken of as the "pellagrin's glove." The manifestations may appear upon the dorsum of the feet even involving the toes, which when classic are known as the "pellagrin's boot." Next in frequency to the hands the process involves the neck usually as a broad band or collar called "Casal's necklace" or "cravat," which has many modifications. The process also occurs in asymmetrical and isolated areas such as the flexion of the elbows, the knees and the axillary folds as well as about the genitalia and the perineum.

The majority of pellagrins are dyspeptic, the dyspepsia being usually of a painful and flatulent type. The alimentary symptoms persist even when the other manifestations of the disease disappear; this is particularly noticeable in the winter during which season the cutaneous symptoms improve and those of the nervous system subside. The mouth, the pharynx, the esophagus, the stomach, and all divisions of the intestines may take part in the pellagrous process. Many observers believe that the gastro-intestinal symptoms are the result of changes in the alimentary mucosa similar to those occurring in the skin, and that as the disease progresses involved areas of the mucosa undergo ulcerative changes. The actual digestive power is frequently good notwithstanding the presence of severe symptoms.

Pellagrins complain of burning sensations in the mouth; the mucous membrane will then always be found to be hyperemic; as the disease progresses other lesions of a stomatitis even to sloughs and bleeding are to be found. The tongue takes part in the pellagrous changes and when it is denuded of its epithelium but not ulcerated, it assumes a peculiar carmin color that is almost pathognomonic. The onset of these conditions in the mouth makes mastication and deglutition painful and difficult. When gastric burning is present it is unaccompanied by any chemical evidence of hyperacidity; repeated examinations of the expressed stomach contents always have shown a low total acidity, no excess of fermentation acids, and the hydrochloric acid content both free and combined to be below normal. The severe attacks of gastralgia that come later in the disease often occur in paroxysms that resemble the gastric crises of tabes, and, sometimes cause vomiting. While constipation is rather frequent early in Pellagra, it is more likely to alternate with diarrhea; the diarrhea occurring early is a spasmodic phenomenon; in grave and fatal cases diarrhea is especially severe and difficult to control. Diarrheas in Pellagra are of two varieties,—one, common early in the disease, is dysenteric in character with colic and with muco-sanguinolent discharges; the other which is present in the later and progressive periods of the disease consists of watery stools that are important factors in producing cachexia. The odor of the bowel discharges is peculiarly offensive probably as the result of putrefactive changes.

Ordinarily the manifestations of Pellagra involving the mind and the nervous system are present when a pellagrin first comes under observation; such are sure to arise during the course of the disease and to have more or less frequent remissions until toward the end when they become continuous and often overtop all other symptoms. Some observers take the stand that all the symptoms in Pellagra of whatever nature are due to some as yet obscure toxic disorder of the brain and nervous system. The psychic manifestations may be ill-defined,—only indicated in the existence of a greater degree of psychic excitability than the normal for the given case. Vague apprehensions, anxiety, unrest, and fears that even may become phobias make up the psychic picture in some cases; in other cases lassitude, depression, mental incapacity, self-depreciation, self-accusation, persecutory ideas and the refusal to take food are the principle psychic features. The patient is often silent with a dull look and a serious countenance which have given rise to the saying,—"He looks as if he had forgotten how to smile."

The first attack of complete insanity supervenes after Pellagra has existed for several years during which several remissions of the skin and the alimentary as well as of the nervous and mental symptoms have taken place. Some observers regard the pellagrin insanities as something apart and different from typical melancholia or typical mania; the attacks seem to pass through two phases,—the first one showing early may be classed as an amentia; the other showing later in time being classed as a dementia. In the very late periods of the disease as it progresses toward fatal termination, a chronic mental confusion, or a ''pellagrous pseudo general paralysis'' may supervene.

Purely mental symptoms may be replaced by manifestations involving the cerebro-spinal nerves or such may be added to the psychic disturbances. Of such are headache, vertigo, meningitis, myelitis, neuralgia, polyneuritis, paresthesia and epilepsy. There usually is present as evidence of spinal irritation,—an increase of tendon reflexes, an increase of muscle excitation with tremors of the fingers and with rigidities and spasms of the leg muscles, a spastic gait, a diminution of the tactile, thermal and faradic cutaneous sensibility, or, an ataxia of the lower and in rare cases of the upper extremities; tonic muscle spasms may be the only nervous symptoms present in a severe Pellagra.

### ETIOLOGY.

The immediate and direct cause of Pellagra has not as yet been determined. It has shown a very marked association with chronic gross alcoholism so much so that the abuse of alcohol is an important predisposing factor. An exposure to a considerable degree of intensity of the actinic rays of the sun, direct or indirect, is an essential for the development of the cutaneous manifestations.

For years a diet consisting chiefly of corn,—maize,—was supposed to be the causal factor in producing Pellagra,—the so-called ''vitamin'' theory. The corn was thought to be lacking in some particular nutritive element necessary to the human economy or else it contained a substance toxic to the human organism,—a substance normally present in the grain or else the result of changes due to more or less decomposition.

In Buffalo, one of the principal ports in the United States for the handling of corn in transit, are located large grain elevators, driers and mills; not only are these used for the transhipment, but also for the handling of grain which has been overheated in the vessels arriving in port,—a quite natural phenomenon taking place in large masses of improperly cured grain, especially of corn. The writer investigated the movement and disposition of the corn received at Buffalo in 1910, and found that twenty millions of bushels of corn had been received, handled and reshipped. Much of this vast quantity arrived in a more or less overheated condition; in the process of cooling and drying with fans such grain was handled several times before it was in a condition fit to leave Buffalo for its destination. The comparatively small amount of corn which was in so damaged a condition as not to be accepted by the consignee, was kiln-dried, ground and sold as chicken feed and hence not used as human food. While some of these millions of bushels of corn was exported most of it went to the New England states for home consumption.

Recalling that much of the corn reaching the port of Buffalo is in a state of at least mild fermentation, and that much of it is subsequently used as an article of human diet, it is interesting to note that in the states to which it is transhipped there has been reported the occurrence of but an occasional sporadic case of Pellagra. On the other hand, an equally interesting observation is that those states reporting the occurrence of large numbers of cases of Pellagra and those states in which the disease is endemic, all receive large consignments of corn from the same sources from which comes that passing through the port of Buffalo. As corn from a common source is freely consumed in both sections and, as Pellagra is practically unknown in the Eastern states while it is prolific in the Southern states, the vitamin theory of the etiology of Pellagra with maize as the dietetic source is not upheld by the experience of the United States.

Another theory that Pellagra is an infection resulting from the introduction into the human body of a specific infective material by the sting of the sand-fly or buffalo-gnat has not been positively demonstrated.

Another theory is that Pellagra results from long continued restricted diet consisting very largely of one kind of carbohydrates. Goldberger and his associates tested this theory by segregating twelve volunteer adult, white, male prisoners at the Mississippi Penitentiary,—wherein Pellagra had never been observed,—and causing them to live on a restricted diet. At the end of six months, the six prisoners who had persisted showed evidences of Pellagra. The skin manifestations first appeared on the scrotum, while both gastro-intestinal and nervous manifestations were noted. As none of the other prisoners living under the same sanitary environments but upon a regular diet developed the disease, the observers concluded that the restricted diet caused the Pellagra that occurred.

Another theory is that Pellagra is the result of drinking water in which there is a relative excess of colloidal silica,—an element largely present in water from clay soils. An enthusiastic supporter of this theory declares that there is no analogy whatever between Pellagra and Beriberi, or between Pellagra and Scurvy; that the vitamin theory is falsely drawn from unscientific observations made to bolster preconceived ideas; that (1) ''Pellagra is a chronic acidosis caused by colloidal silica in drinking water coming from clay soils,'' that (2) ''Pellagra is a disease strictly localized and contracted only in those regions where the water commonly drunk originates in clay soils,'' and, that (3) ''Pellagra has no relation to diet, work, domicile or sanitary environment.''

### PATHOLOGY.

The postmortem findings in cases dead with acute Pellagra are in general those produced by an acute poisoning. The perivascular infiltration indicates general systemic intoxication. There is an indirect chromatolysis of nerve cells, astrocytosis, and, the presence of ameboid glia cells. No evidence of infection of the nervous system by microorganisms has been found. The autopsy in cases with chronic Pellagra shows no definite pathology that can be attributed solely to Pellagra; although there have been noted fatty and fibroid degenerations, chronic alterations in nerve cells, an increase of glia fibers, a permanent destruction of nerve fibers, and, a marked increase of amyloid bodies.

The pathological findings in the intestinal mucosa have been those of hyperemia, while ulcerations were not uncommon. The other viscera have shown chronic, particularly fatty, degenerative changes with marked pigmentation. The pathological changes in the skin present nothing characteristic; they are chiefly those of inflammation. The epidermis is at times thinner than normal while at other times it is thickened with considerable hyperkeratosis; there is a pronounced increase of pigment in the lower cells of the reta. In cases having repeated attacks of dermatitis there has been found atrophy of the corium, of the epidermis, and of the nerves of the corium of such permanent character that it may be considered as secondary. The nerves of the skin have been found to exhibit evidence of a combined inflammatory and degenerative process with the disappearance of many axiscylinders.

### CASE HISTORIES.

Case No. 1. (Buffalo Medical Journal, May, 1911). The first indigenous case of Pellagra in Buffalo. E., white housewife; aged 33; born and always lived in Buffalo. Mother of three children. Manifestations first noted in 1906, a few months after birth of last child and a few weeks after a pelvic operation. Nothing in the family or the personal history, habits, environment or diet to suggest Pellagra as the cause of the patient's symptoms. First noted undue fatigue from exertion, marked weakness of legs causing unusual gait and finally simulating paralysis. Later, mental depression appeared ending in an attack of acute mania which lasted but a short time; this was succeeded by great depression. Although the tongue often showed the characteristic scarlet-red appearance the gastro-intestinal manifestations and diarrhea came late in the course of the disease. The skin manifestations present when referred to the author came on about four months preceding her death in 1910; the dermatitis began as a marked erythema with subsequent scaliness, thickening and pigmentation; the almost pathognomonic symmetrical, triangular distribution on the backs of the hands and wrists was easily distinguished from other cutaneous diseases and made the diagnosis positive. No autopsy was obtained.

Case No. 2. (Buffalo Medical Journal, April, 1915), by Spangenthal. L. K., single, druggist, white, male; aged 35; born in Pennsylvania and lived in a small northern Pennsylvania town. Family and personal history negative. Used alcohol in great excess especially just preceding observation; habits otherwise fair; nothing otherwise unusual or indicative in environment or diet. When first seen his mental condition was fair with the exception that he worried greatly over trifles. Gastro-intestinal manifestations were such as are found in alcoholics. Consulted Dr. Spangenthal in the Spring of 1915 for skin trouble at which time Pellagra was recognized and the diagnosis made. The buccal surfaces showed the usual bright-red appearance and at a little later date the diarrhea became manifest. The backs of the hands presented symmetrical bleb lesions which upon rupture in a short time left marked crusting, followed later by the pigmentation characteristic of the disease. After the administration of salvarsan there was an apparent improvement which subsequent events demonstrated to be the result of the stoppage of the ingestion of the large quantities of alcohol. He continued greatly improved for a period of one year after which he began to drink in excess which brought back a marked cutaneous outbreak accompanied by diarrhea. He lived for about two years after coming under observation. No autopsy.

Case No. 3. H., single, saloon-helper; white, female; aged 24; born and always lived in Tonawanda, New York. Family and personal history negative except that the patient had had periodical attacks of diarrhea for the year preceding her entrance to the Buffalo General Hospital, June 1, 1913, and also had had a poor appetite but as she was a large imbiber of alcohol such symptoms might not be unusual.

General examination on admission demonstrated a fair condition with temperature of 102°, pulse rate of 110 and respirations 32; there was a congenital malformation of the digits of both hands. The patient was very emotional showing rapid changes from violent weeping to outbursts of temper. Later she became melancholic and toward the end became comatose. She swallowed with difficulty and her oral mucous membranes were intensely red and ulcerated; the same condition was found in the rectum and the vagina. The skin manifestations upon which the diagnosis was made were characteristic of Pellagra and were symmetrically placed upon the backs of both hands, the forearms, the face and the feet, while about the neck was a faint ''Casal's necklace.'' Upon the backs of the hands were patches denuded of epithelium, in which were considerable sized irregular areas containing many ecchymoses and covered with a serous exudate. Upon the face especially on the nose and adjacent areas was found a marked condition of comedones.

Examination of the blood showed the red cells to number 5,020,000, the white cells 7,200, and the hemoglobin to be 65%; the differential leucocytes count showed the lymphocytes to be 71%; the large mononuclears, 15% and the polynuclear neutrophiles, 64%.

The patient died about two weeks after entrance to the hospital, the autopsy showing: The lungs to be pneumonic, the exudate in the vesicles to consist of leucocytes and desquamated epithelial cells; the heart to have a marked increase of small cell infiltration especially on the pericardial surface; the liver to be somewhat fatty, infiltrated and to be particularly infiltrated with round cells; the kidney cells to have marked cloudy swelling and the vessels to be somewhat sclerosed; the splenic vessels to be moderately thickened; and the intestinal tract, especially the colon to have a markedly degenerated and ulcerated mucosa some of which ulcers extended down to the peritoneal coat; the ulcers varied in size from that of a lead pencil to that of a ten-cent piece, and were irregular and ragged in outline.

Case No. 4. A. C., Coudersport, Pa., white American housewife; multipara; aged 25. Born in the United States. The family history was negative. The personal history related the occurrence of the usual diseases of childhood including scarlet fever, also typhoid fever and malaria. At three years of age she accidently drank lye, since which she claims to have been unable to eat ''solid meats.'' In 1905 at age 17, she had a vesicular eruption on the neck. Her menstruation, beginning at age 14, always had been regular except for the last few months, there having been none for three months. She disclaimed having any venereal disease; there was no disturbance of urination. The patient had always slept well; her appetite had been excellent until recently; her diet had contained nothing unusual either as to quality or quantity. For the past four years she had taken brandy in large quantities and particularly so during the past few weeks. Her surroundings had always been fair; during the month of June 1913 the patient had been constantly out of doors lying in a hammock.

The patient was admitted to the Buffalo General Hospital on July 7th, 1913, in a morphine stupor with the statement that she had been mentally depressed for the past four weeks, and very melancholic for the past two weeks. On the day admitted she had suddenly developed an unmanageable raving requiring morphine to control. She had a skin disease on both arms that first appeared about June 1st, 1913, as a drying and a cracking of the skin, followed later by a moist rawness; about the same time the tongue and lips became sore.

Upon examination July 9th, 1913, the patient kept up an incessant muttering and yet was able to answer questions; there was a marked retraction of the head with now and then an opisthotonos of several minutes; again there would be a seizure showing both tonic and clonic spasms; there were present subsultus tendinum, wrist drop, and delayed tendon reflexes. The mento-nervous condition became one of increasing apathy, dullness and stupor with coma vigil until death supervened on July 10th, 1913.

The temperature on admission was 101; the pulse, 110; the respirations, 30; the maximum blood pressure, 135. The temperature rapidly climbed to 104, the pulse to 125 and the respirations to 55 just before death. The heart was normal; the lungs showed signs of massive pneumonia so that a diagnosis of double lobar pneumonia was made. The pupils were equal and contracted but reacted to light; the conjunctival vessels were engorged. The cervical lymph glands were palpable.

The alimentary manifestations were confined to the oral cavity and were so severe as to make it difficult to open the mouth. The lips were cracked and covered with crusts; the gums showed white ulcerations at base of each tooth. The tongue was a moist red and showed many white pea-sized patches of ulceration; it could be only slightly protruded; it was markedly tremulous and had a heavy white coating. The buccal mucosa showed a general stomatitis while the voice was a husky whisper. There were no acute gastric or intestinal symptoms or signs, nor to any of the other abdominal viscera. Along the labia minora were found many pin-head to pea-sized growths and irregular whitish spots; the vaginal mucosa was covered with small whitish spots and there was a foul purulent leucorrhea.

The skin manifestations were typically characteristic and upon them the diagnosis of acute Pellagra was made. The lesions were symmetrical, irregular areas of dermatitis, the edges of which were vermicular in conformation. The face had a tanned hue while areas of dermatitis were located on the upper eyelids at the nasal side. Upon the extensor surfaces of both forearms, upon the backs of both hands as far as the proximal phalangeal joints, upon the backs of both wrists extending around on the radial side to the midline on the flexor surfaces, in the right antecubital fossa for the size of a silver dollar and in the left for the size of a silver quarter, were the characteristic dry scaly patches of grayish to blackish color. Where desquamation had taken place the underlying color was of a violaceous red. In some of the denuded areas small hemorrhages had occurred with the formation of small hardened blood clots; while along the edges of the lesions, in areas of previous involvement and especially in those on the flexor surfaces were found deposits of brown pigment the outlines of which were gyrated, surrounding centers of lack of pigment.

In the laboratory investigations the urine otherwise negative showed the presence of traces of albumen and the presence of pus cells. The blood showed a hemoglobin content of 82% by the Sahli instrument; the red cells numbered 4,600,000, the white cells 7,800; the differential white cell count showed polynuclears 75%, small lymphocytes 10%, large lymphocytes 7%, eosinophiles 8%. The Wassermann tests of both the blood serum and the spinal fluid were negative. The spinal fluid pressure was slightly under 5 m.m. No autopsy.

Case No. 5. T. H. K., male, white American, widower, farmer, aged 77. Born in Central New York State, resided in Buffalo during the four years last past coming to Buffalo from a residence of several years in Pittsburgh, Pa.; with this exception he spent most of his life near Buffalo; he has been in Tampa, Fla. The family and personal history obtained was quite irrelevant on account of mental state of patient. Friends stated that his present mental and physical condition began in 1908 at age of 70. His facial expression grew steadily duller so that his face assumed the mask-like state seen when he came under observation. His voice gradually had taken on a high pitched monotonous quaver often ending as an inarticulate mumbling. His mental reaction time had become so slow that he was often many seconds in replying to simple questions which if repeated caused him to become angry and to scold. He gradually had lost orientation. He slowly had become querulous, suspicious and morose while at times he was excited and delirious. He would often refuse food and remedies except whiskey. During the few days preceding admission he was more or less comatose. For the same reason it was impossible to ascertain about environment, habits or past diets, although there was nothing to indicate anything out of the ordinary for a well-to-do American. The patient entered the Buffalo General Hospital June 25th, 1914, complaining of itching and dropsy. On admission the patient's temperature by mouth was 100, and, his pulse was 120.

Examination showed the patient's lungs to be normal; the heart sounds to be weak otherwise normal. There was some ptosis of the upper eyelids; the conjunctivae were inflamed and the pupils were sluggish to light. Reflexes responded to stimuli. The mental state was one of deep apathy even of coma; he was irritable upon being aroused or examined. The teeth were poor; there was a mild stomatitis with slight salivation; the oral mucosa was of a bright-red hue. While no direct complaint of the stomach was made, there were some vague gastric symptoms; the patient had frequent fecal evacuations. The skin manifestations were very pronounced and characteristic being bilateral, symmetrical and sharply defined. They were present on the hands, in the precubital fossae, on the face, on the neck and about the genitalia. There was a dollar-sized necrotic area on the right buttock. The lesions on the backs of the hands were desquamating and showed a deep violaceous-red color; they extended from the finger tips to the wrists; the palms were unaffected, while the anterior surfaces of the wrists showed the characteristic V-shaped patches with the apex pointing toward the elbows. The areas in the bends of the elbows were diamond-shaped of a dull-red color with the acute angles pointing up and down the arm while the sharply defined edges were desquamating. The neck was involved with similar appearing patches forming the so-called collarette. The lesions on the face extended to the scalp as blotches; they were dull-red violaceous desquamating patches of chronic dermatitis which gave a mask-like expression to the face.

The urine was negative except for a marked indicanuria. The Wassermann blood test was negative. The patient did not rally but gradually grew weaker and died about July 14th, 1914. No autopsy.

Case No. 6. Reported through the courtesy of Dr. Arthur W. Hurd, Superintendent of the Buffalo State Hospital, to whom I am indebted for the history. H. O., Polish nun, never married, aged 35. Born in Poland, emigrated to the United States in 1891 when eleven years old and settled in Buffalo; she entered the order of the Sisters of Charity in 1899 at the age of nineteen; she was transferred to a mission in Massachusetts where she lived for two and one-half years when she returned to Buffalo; a few months thereafter she was sent to a mission in Pennsylvania from which she returned to Buffalo in April 1905, where she has lived ever since. The family history indicates degeneracy; the father, a common laborer, deserted the family, the mother had manic depressive insanity and at least one sister had dementia praecox.

The personal history is silent in regard to general diseases. The patient never was very bright and she was backward in her studies; she was always thought "peculiar." In 1903, at age 21, she was sent to Providence Retreat on account of pronounced mental disturbances which so cleared in eight months that she was permitted to leave. On May 16th, 1905, she was sent to the institution as an insane person; during this incarceration depressed, melancholic periods followed others of violent excitement and led to a diagnosis of dementia praecox; she so improved that she was paroled in May 1906. She again was admitted on August 10th, 1911, and has remained an inmate ever since; her residence has been marked by fluctuations of depression and excitement in her mental state. That she also has nerve disturbances is indicated by her complaints of headaches and of pain in right arm and fingers at various times; by the wrapping up of her wrists, and by the wearing of extra clothing because of sensations of coldness. The nervo-mental symptoms seem always to have been worse during the late summer and the early fall seasons. The patient was habitually constipated; she took an abundance of food and was considered to be quite obese; corn was never an especial item in her diet; she was usually a voracious eater even gleaning from the garbage as opportunity offered. There was nothing to indicate that the early or late environment of the patient had been such as to influence the appearance of the manifestations except that she habitually and for hours so sat that her left side was exposed to the direct rays of the sun.

Late in May 1915, reddened areas resembling burns were noted upon the back of the left hand and wrist but they did not clear up. In June 1915 a scratch-like red mark came under the left eye, which by the middle of July 1915 had spread over to the left side of the nose; later other areas appeared on the left cheek, on the left forehead at the hair line, on the left side of the chin, on the left side of the neck and on the left ear.

A Wassermann blood test made January 20th, 1915, proved to be negative.

An examination made July 16th, 1915, showed the patient to be in good flesh; that there was a slight elevation of temperature, 99.2 per rectum in the afternoon, an accelerated pulse and a normal respiration; and the urinalysis to be negative. Her mental condition was one of melancholy; she was depressed, wept easily, and refused to answer questions.

The mouth, the tongue and the pharynx presented nothing abnormal; there was no complaint about the stomach or bowels except of a chronic condition of constipation; no suggestion of diarrheal attacks was obtained.

Although the skin manifestations upon which the diagnosis of Pellagra was made were more pronounced upon the left side nevertheless they were symmetrically placed; this difference was due to the more intense exposure of the left side to the sunlight. Areas of varying size involved not only the backs of both hands from the finger tips to the wrists, but also both sides of the face and the neck; the demarcation lines were sharply defined from the normal skin; the older ones presented vesicles and crusts, and more or less thickening, scaling and desquamation; a large bleb covered the left wrist; the color of the lesions was of a violaceous hue. By October 1st, 1915, the skin manifestations had disappeared. The entire picture presented by the skin demonstrated the characteristic evolution of the dermatitis peculiar to Pellagra.

Case No. 7. M., married, male Jew, storekeeper, aged 54; born in Germany but lived in Elmira, New York, for more than the past forty years. Habits, good. Came under observation July 1st, 1915.

The family history contained nothing pertinent; the personal history was negative except that more or less continuously for a number of years back he had suffered from insomnia, weakness and vertigo, while recently he had had skin disturbances. Examination showed the tongue to be heavily coated but not typically pellagrous. The cutaneous manifestations were typical in that the backs of both hands were symmetrically involved from the tips of the fingers to their junction with the wrists; the entire area was wrinkled, thickened, rough and covered with firm adherent scales; the color was a dirty or blackish gray. The margins of the lesions were sharply defined. The flexor aspects of the wrists showed less pronounced triangular areas with the apices on the radial side pointing toward the elbow. The body presented no other cutaneous manifestations. There never had occurred any marked or disturbing symptoms referable to the brain and nervous system or to the alimentary tract except as stated above.

In a personal report from the patient January 1st, 1918, he stated that he had fully recovered, in fact was in better health than at any time for a number of years. The treatment continued for one year consisted of the hypodermic administration of cacodylate of soda alternating with quinine. This case possessed enough of the characteristic features of Pellagra to warrant the diagnosis and its corroboration by Dr. W. A. Pusey.

Case No. 8. E. P., single, white, female, school teacher, aged 23; born of American parents in Brocton, N. Y., in which place she always lived with the exception of one year as a teacher in Easthampton, L. I. The family history was negative. The personal history recorded the usual diseases of childhood; menstruation came on at 13, and became irregular during the year 1915. In 1905, when 12 years of age and while spending a summer month at Allegheny Springs, Pa., she had an attack of diarrhea which persisted for some time after her return home; no similar attack has recurred. In the summer of 1907 she had an attack of what she called "inflammation of the stomach." In the fall of 1915 she was confined to her bed for several days with something not diagnosed but which was accompanied by "high fever." The environment of the patient had always been of the best; she always had had a generous and varied diet into which corn entered but very little as the patient did not care for it; her weight had been fairly constant but with a seasonable variation,—105 pounds in the summer to 112 pounds in the winter.

In June 1916, she sought relief from an eruption on the skin which first invaded the back of the neck and then the arms.

Upon examination July 28th, 1916, the patient was found to be under size and light weight; to present assymetries of eyes, teeth and palate and to have the general type of an hysterical individual; she gave no history of headaches or neuralgias; she was emotional, easily disturbed but showed no active depression. The tongue was enlarged on the left side and looked as though the epithelial layer had been removed; the buccal mucous membrane was red and inflamed.

The patient also complained of vomiting and of periodic attacks of diarrhea followed by constipation.

The neck was encircled by an erythematous and scaly chronic dermatitis forming a collarette. The dorsal surfaces of both hands showed a chronic, thickened erythematous dermatitis; the dermatitis did not end abruptly at the wrists but extended in large patches over the extensor surfaces of the forearms and arms; the pigmentation of the areas about the finger joints was more intense than elsewhere on the hands and the arms; the borders of the patches on the arms and forearms were well defined; the scaling varied somewhat in amount.

The blood examination demonstrated the hemoglobin to be 70% (Fleischal) the red cells 3,600,000; the white cells 10,-400; the differential count to be polynuclear cells 64%, the small lymphocytes 23%, the large lymphocytes 3%, the mononuclears 5% and the eosinophiles 5%.

In August 1916, the patient was committed to a hospital for the insane on account of a sudden onset of maniacal insanity. Subsequent history unknown.

(To be continued in July issue.)

---

**Enterococcic Infections.** Tricoire, Le Prog. Med., Nov. 17, 1917, describes the cultural identification. This germ, along with the b. coli and the b. bifidus, is found normally in the intestine of man and most of the domestic animals and hence, commonly, in the soil and water, and adventitiously on the skin and invarious excretions. It may become pathogenic, causing entero-colitis or gastro-enteritis, especially in nurslings but may also invade the extensions of the digestive tube or may extend from it in other directions, so as to cause a great variety of lesions, as peritonitis, inflammations of the biliary passages, hepatic abscess, may accidentally invade the urethra, like the colon bacillus, may cause broncho-pneumonia, appear in the guise of rheumatism of all types, arthritic, endocardial and muscular, of meningitis, or skin affections. It may also take the form of a bacteriaemia without definite localization, and produce continued or intermittent fever. The seriousness of the infection varies greatly, being marked in the meningeal type or when the lungs or joints and endocardium are involved, usually mild when the alimentary canal reacts, unless, of course, there is extension into the substance of the liver. Aside from general treatment, the production of an ''abscess of fixation'' or vaccinotherapy is suggested, but apparently only on general principles.

